# Inhibition of Aflatoxin Production in *Aspergillus flavus* by a *Klebsiella* sp. and Its Metabolite Cyclo(l-Ala-Gly)

**DOI:** 10.3390/toxins16030141

**Published:** 2024-03-08

**Authors:** Shohei Sakuda, Masaki Sunaoka, Maho Terada, Ayaka Sakoda, Natsumi Ishijima, Noriko Hakoshima, Kenichi Uchida, Hirofumi Enomoto, Tomohiro Furukawa

**Affiliations:** 1 Department of Biosciences, Teikyo University, 1-1 Toyosatodai, Utsunomiya 320-8551, Japanhakoshima@nasu.bio.teikyo-u.ac.jp (N.H.); kuchida@nasu.bio.teikyo-u.ac.jp (K.U.); enomoto@nasu.bio.teikyo-u.ac.jp (H.E.); 2 Institute of Food Research, National Agriculture and Food Research Organization, 2-1-12 Kannon-dai, Tsukuba-shi 305-8642, Japan; furukawat795@naro.affrc.go.jp

**Keywords:** biocontrol, aflatoxin production inhibitor, *Klebsiella*, diketopiperazine, cyclo(l-Ala-Gly)

## Abstract

During an experiment where we were cultivating aflatoxigenic *Aspergillus flavus* on peanuts, we accidentally discovered that a bacterium adhering to the peanut strongly inhibited aflatoxin (AF) production by *A. flavus*. The bacterium, isolated and identified as *Klebsiella aerogenes*, was found to produce an AF production inhibitor. Cyclo(l-Ala-Gly), isolated from the bacterial culture supernatant, was the main active component. The aflatoxin production-inhibitory activity of cyclo(l-Ala-Gly) has not been reported. Cyclo(l-Ala-Gly) inhibited AF production in *A. flavus* without affecting its fungal growth in a liquid medium with stronger potency than cyclo(l-Ala-l-Pro). Cyclo(l-Ala-Gly) has the strongest AF production-inhibitory activity among known AF production-inhibitory diketopiperazines. Related compounds in which the methyl moiety in cyclo(l-Ala-Gly) is replaced by ethyl, propyl, or isopropyl have shown much stronger activity than cyclo(l-Ala-Gly). Cyclo(l-Ala-Gly) did not inhibit recombinant glutathione-*S*-transferase (GST) in *A. flavus*, unlike (l-Ala-l-Pro), which showed that the inhibition of GST was not responsible for the AF production-inhibition of cyclo(l-Ala-Gly). When *A. flavus* was cultured on peanuts dipped for a short period of time in a dilution series bacterial culture broth, AF production in the peanuts was strongly inhibited, even at a 1 × 10^4^-fold dilution. This strong inhibitory activity suggests that the bacterium is a candidate for an effective biocontrol agent for AF control.

## 1. Introduction

Some *Aspergillus* sp., such as *Aspergillus flavus* and *Aspergillus parasiticus*, infect food products and contaminate them with aflatoxins (AFs), which are fungal secondary metabolites with high toxicity and strong carcinogenicity [1,2]. AF contamination in food and feed affects human and animal health, and it has a serious impact on the agricultural economy [3]. Although AF contamination in crops occurs in tropical and subtropical areas, 4.5 billion people are potentially exposed to AFs through global food distribution [4,5]. As a large amount of food contaminated with AFs is discarded because of regulations in many countries, preventing AF contamination is necessary for protecting human health and resolving food shortages [6]. However, few methods are currently available for preventing AF contamination; therefore, it is critical to develop effective and practical methods for this purpose.

Although the use of antifungal agents is a possible method for controlling AF contamination [7], their use can lead to the emergence of strains resistant to antifungal agents used in AF control and medicine. Because AFs are fungal secondary metabolites that are not necessary for fungal growth, AF production inhibitors that do not affect fungal growth can be used for AF control without incurring the rapid spread of resistant strains [8]. Inhibitors that specifically target AF production are also useful biological probes for investigating the AF production mechanism in fungi, which is important for developing novel AF control methods. Therefore, we have been studying AF production inhibitors obtained from microbial metabolites, essential oils, pesticides, and food additives and investigating their modes of action in inhibiting AF production [8,9].

Fungi and bacteria have been assessed as biocontrol agents which are friendlier to the environment than chemical pesticides in preventing AF contamination. Non-aflatoxigenic strains of *Aspergillus* that can competitively exclude aflatoxigenic strains from crops are used in practice for AF control [10,11]. However, with this method, safety issues, such as the production of other toxic metabolites by the non-aflatoxigenic strain, have not been eliminated [12,13]. Many microbes have displayed potential for AF control, and they can be classified into the following groups: group 1, which can inhibit fungal growth and, consequently, AF production; group 2, which can inhibit AF production without affecting fungal growth; group 3, which can inhibit AF production and produce an AF production inhibitor; group 4, which can degrade AFs; and group 5, which can absorb AFs on their cells. Excellent review articles on these AF control microbes have recently been published [14,15]. We have been studying the group 3 microbes and found that *Stenotrophomonas* sp. can inhibit AF production by *A. flavus* and *A. parasiticus* and produce the AF production-inhibiting diketopiperazines cyclo(l-Val-l-Pro) and cyclo(l-Ala-l-Pro) [16]. Cyclo(l-Leu-l-Pro), a similar diketopiperazine that inhibits AF production, was isolated from the group 3 microbe *Achromobacter xylosoxidans* [17]. The AF production-inhibiting diketopiperazines isolated from group 3 microbes have commonly contained l-proline residues.

Recently, during an experiment in which aflatoxigenic *A. flavus* was cultivated on peanuts, a bacterium adhering to the peanuts was found to inhibit AF production by the fungus. The bacterium, designated strain KTTM, was found to produce an inhibitor of AF production. Here, we describe the isolation and identification of the strain KTTM and the AF production inhibitor produced by the strain, as well as the effects of the inhibitor, its related compounds, and the bacterial cells on AF production. The inhibitor was identified as a diketopiperazine without the L-proline residue, and the cells exhibited a strong inhibitory activity of AF production by *A. flavus* cultivated on peanuts.

## 2. Results

### 2.1. Isolation and Identification of the Strain KTTM

The strain KTTM was discovered from peanut paste in an experiment conducted in another study. To investigate the AF production of *A. flavus* in peanut paste, peanuts were ground in a grinder, dispensed into the wells of a 12-well plate, and inoculated with *A. flavus*. After several days, we found that fungal growth in one well was severely inhibited. A dilution series with water was prepared from the paste of this well and spread on agar plates. Several single colonies were obtained, and each bacterium involved in a colony was tested for AF production-inhibitory activity by co-culturing *A. flavus* with the bacterium in a liquid culture. A bacterium that inhibited the AF production was designated as strain KTTM, which was a rod-shaped, Gram-negative bacterium, and it was identified as *Klebsiella aerogenes* by morphological and biochemical analysis as well as by a comparison of its 16S rDNA sequence with those in a database (99.6% identity with *Klebsiella aerogenes* NBRC 13534 [AB680425]; Figure S1).

### 2.2. Isolation and Identification of the AF Production Inhibitor Produced by the Strain KTTM

The culture supernatant of the KTTM strain was applied to a charcoal column packed with water and eluted with 10% ethanol after washing the column with water. AF production inhibition, which was tested in a liquid medium inoculated with *A. flavus*, was observed in the 10% ethanol eluate. The 10% ethanol eluate fraction was purified twice by HPLC using a C_18_ column and further purified by HPLC using a C_22_ column to obtain the active component.

**Figure 1:**
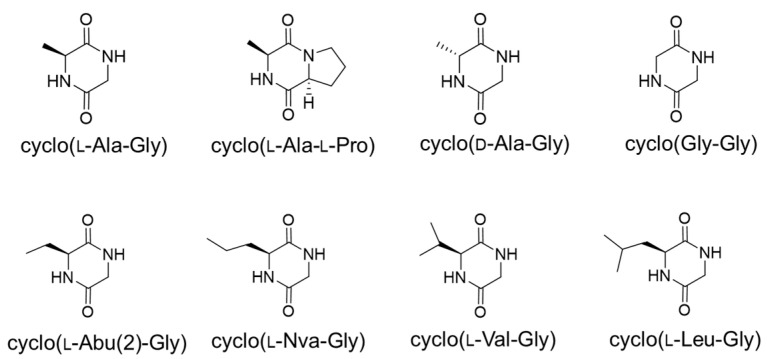
Structures of cyclo(l-Ala-Gly) and related compounds.

The ^1^H and ^13^C NMR spectra of the active component (Figures S2 and S3) suggested the presence of one residue each of Ala and Gly in it, and its molecular formula was determined as C_5_H_8_N_2_O_2_ based on the HRESI-Q/TOF mass spectrum. These findings strongly indicated that the active component was a diketopiperazine consisting of Ala and Gly. A comparison of the optical rotation value and the retention time on HPLC with those of commercial cyclo(l-Ala-Gly) identified the active component as cyclo(l-Ala-Gly) (Figure 1).

### 2.3. AF Production Inhibition by Cyclo(l-Ala-Gly) and Related Compounds

**Figure 2:**
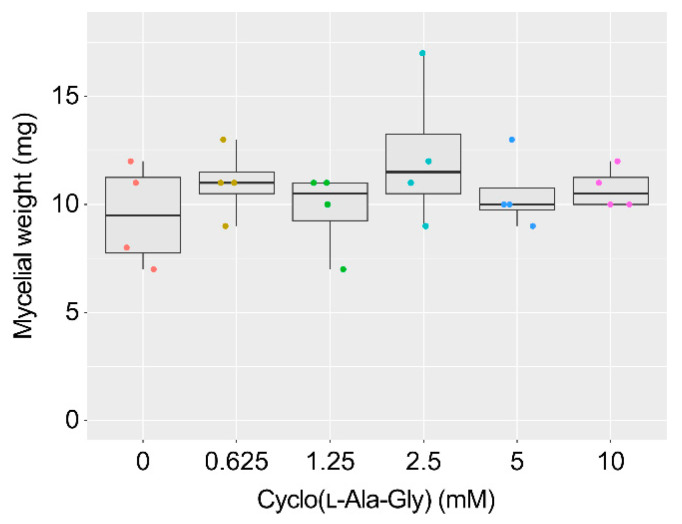
Effect of cyclo(l-Ala-Gly) on the mycelial weight of *A. flavus.* Boxplots of the dried mycelial weight. The colored dots indicate individual values. *n* = 4.

Cyclo(l-Ala-Gly) inhibited aflatoxins B_1_ (AFB_1_) production by *A. flavus* with an IC_50_ value of 0.75 mM in a liquid culture (Table 1). The compound did not affect fungal mycelial weight even at a concentration of 10 mM (Figure 2). The inhibitory activity of cyclo(l-Ala-Gly) was stronger than that of cyclo(l-Ala-L-Pro), which displayed the strongest activity among the three known l-Pro-containing diketopiperazines. Cyclo(d-Ala-Gly) exhibited no inhibitory activity at 10 mM, indicating the importance of the l stereochemistry of the Ala residue to the inhibition activity.

**Figure 3:**
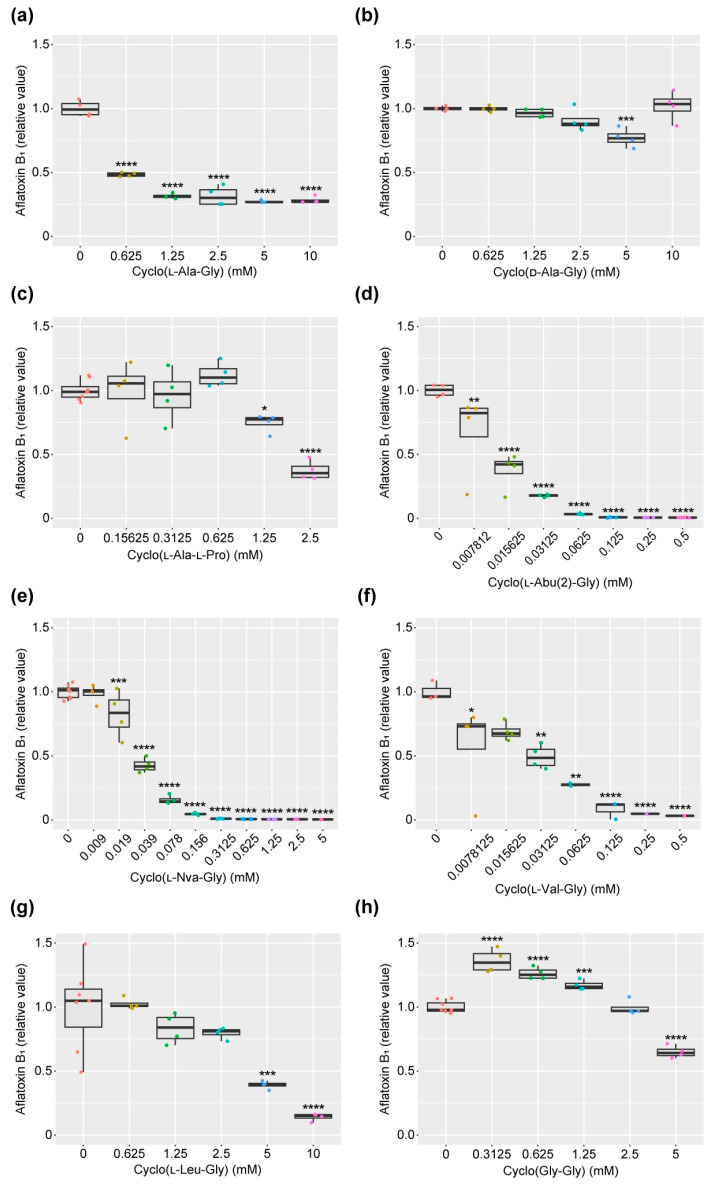
Aflatoxin production-inhibitory activity of cyclo(l-Ala-Gly) and related compounds. Boxplots of the relative production levels of aflatoxin B_1_. The colored dots indicate individual values. *n* = 4–8. * *p* < 0.05, ** *p* < 0.01, *** *p* < 0.001, and **** *p* < 0.0001 versus no added control, ordinary one-way ANOVA followed by Dunnett’s test. (**a**) cyclo(l-Ala-Gly), (**b**) cyclo(d-Ala-Gly), (**c**) cyclo(l-Ala-l-Pro), (**d**) cyclo(l-Abu(2)-Gly), (**e**) cyclo(l-Nva-Gly), (**f**) cyclo(l-Val-Gly), (**g**) cyclo(l-Leu-Gly), and (**h**) cyclo(Gly-Gly).

Five compounds similar to cyclo(l-Ala-Gly), namely, cyclo(l-Abu(2)-Gly), cyclo(Gly-Gly), cyclo(l-Val-Gly), cyclo(l-Nva-Gly), and cyclo(l-Leu-Gly) (Figure 1), were prepared to obtain information on the structure–activity relationship of the methyl side-chain moiety in cyclo(L-Ala-Gly). As shown in Table 1, summarizing the IC_50_ values deduced from the data in Figure 3, the AF production-inhibitory activity was dramatically changed by the structure of the side chain. Cyclo(l-Abu(2)-Gly), cyclo(l-Val-Gly), and cyclo(l-Nva-Gly), in which the methyl group of cyclo(l-Ala-Gly) was replaced by ethyl, isopropyl, and propyl, respectively, more strongly inhibited AF production (75-, 18.8-, and 8.3-fold stronger, respectively) than cyclo(l-Ala-Gly). However, cyclo(l-Leu-Gly), possessing an isobutyl group, exhibited weaker activity than cyclo(l-Ala-Gly). Cyclo(Gly-Gly), lacking side chains, displayed weak activity at 5 mM (Figure 3).

### 2.4. Inhibition of AF Production by the Strain KTTM

**Figure 4:**
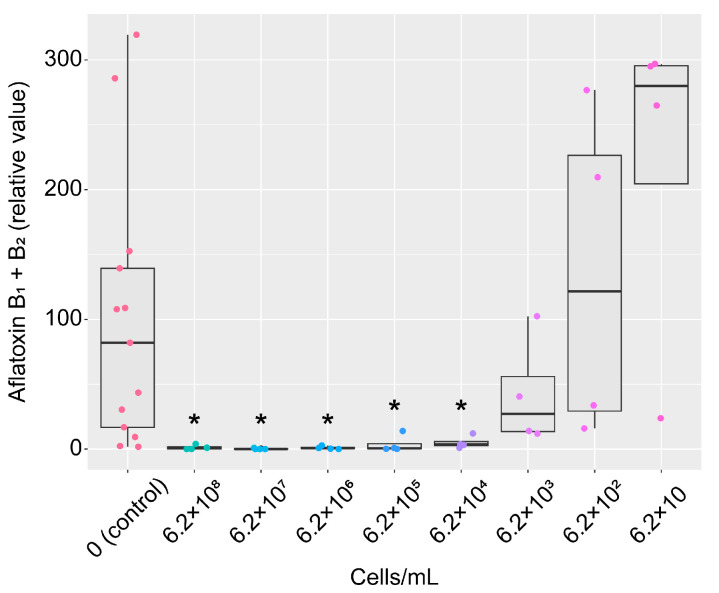
Effects of the strain KTTM on AF production by *A. flavus* grown on peanuts. Boxplots of the relative amounts of aflatoxins B_1_ and B_2_. The mean value of AF amounts in the 0 (control) group was set as 100. The colored dots indicate individual values. *n* = 4 (treated) or 13 (control). * *p* < 0.05 versus control, Brown–Forsythe and Welch ANOVA followed by Dunnett’s T3 test.

The effect of the KTTM strain on AF production was tested by cultivating *A. flavus* on peanuts. After autoclaving law peanuts lacking shells and skins, each peanut was shortly dipped in the strain KTTM culture broth or a dilution of the culture broth and inoculated with *A. flavus* spores. After 30 days of incubation at 25 °C, the amount of AF in each peanut was measured. AF production was significantly reduced even upon exposure to 1 × 10^4^-fold diluted broth (6.2 × 10^4^ cells/mL) without affecting the growth of *A. flavus* (Figure 4 and Figure S4).

### 2.5. AF Degradation Activity of the Strain KTTM

**Figure 5:**
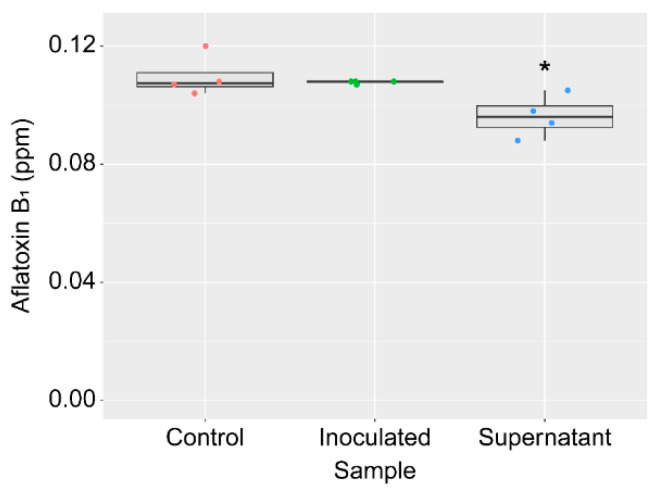
AF degradation activity of the strain KTTM. Boxplots of the AFB_1_ amounts. The colored dots indicate individual values. *n* = 4. * *p* < 0.05 versus control, ordinary one-way ANOVA followed by Dunnett’s test.

As a report has demonstrated that *Klebsiella* sp. degrades AF [18], the AF degradation activity of the strain KTTM was examined. When AFB_1_ was added to a liquid medium and the strain KTTM was cultured in the medium for 3 days, the concentration of AFB_1_ in the culture broth was consistent with that of the control in which AFB_1_ was kept in the liquid medium without inoculation of the bacterium for 3 days (Figure 5). By contrast, when AFB_1_ was incubated in the KTTM strain supernatant of the culture broth, its concentration was slightly lower than that of the control (Figure 5).

### 2.6. Effects of Cyclo(l-Ala-Gly) and Related Compounds on A. flavus Glutathione-S-Transferase Activity

We identified *A. flavus* glutathione-*S*-transferase (AfGST) as a binding protein of cyclo(l-Ala-l-Pro) and found that the compound inhibited AfGST activity [19]. To investigate the mode of action of cyclo(L-Ala-Gly), the effects of cyclo(l-Ala-Gly) and its related compounds on AfGST activity were evaluated using bacterially expressed AfGST and 1-chloro-2,4-dinitrobenzene as the substrate. The known AfGST inhibitors, cyclo(l-Ala-L-Pro) and ethacrynic acid, were used as positive controls. Three diketopiperazines with weak or no inhibitory activity against AF production, namely, cyclo(d-Ala-Gly), cyclo(l-Leu-Gly), and cyclo(Gly-Gly), weakly inhibited AfGST activity (Figure S5). However, four diketopiperazines that strongly inhibited AF production, namely, cyclo(l-Ala-Gly), cyclo(l-Abu(2)-Gly), cyclo(l-Val-Gly), and cyclo(l-Nva-Gly), did not inhibit AfGST activity at the concentrations tested (Figure S5).

## 3. Discussion

We found a bacterium, named strain KTTM, with strong AF production-inhibitory activity by chance. The strain was identified as *K. aerogenes,* and it had a high 16S rDNA sequence homology with *Klebsiella aerogenes* NBRC 13534. The KTTM strain culture broth effectively inhibited AF production by *A. flavus* even after being diluted 1 × 10^4^-fold, suggesting that a mass number of bacterial cells is not necessary for AF control when the strain is used in practice as a biocontrol agent.

The diketopiperazine cyclo(l-Ala-Gly) was isolated from the culture supernatant of the KTTM strain as an AF production inhibitor. The diketopiperazines produced by microbes can be classified into two groups, namely, one group with simple structures consisting of two amino acids and another group with relatively complex structures biosynthesized by modifying the simple diketopiperazines of the first group [20]. The latter group includes many secondary metabolites with a variety of bioactivities, and they are produced mainly by fungi and Actinobacteria. Concerning the former group, many diketopiperazines, such as cyclo(l-Ala-Gly), are metabolites of a wide range of microbes [21,22]. Although some diketopiperazines of the former group exhibit bioactivities such as antimicrobial activity [23], their bioactivities are less specific and weaker than those of the latter group. Therefore, there are few in-depth studies on the bioactivities of the former group of diketopiperazines, excluding those that inhibit AF production. Cyclo(l-Ala-Gly) and related compounds, which were found to inhibit AF production in this study, could be useful as both lead compounds for developing AF control agents and as biological probes for investigating the regulatory mechanism of AF production in aflatoxigenic fungi.

The culture supernatant of two *Klebsiella* sp. strains was reported to degrade AF [18], as the amount of AF decreased by approximately 70% after incubation with the culture supernatant for 3 days. Compared to the activity of the reported strains, that of the strain KTTM was weak. As the amount of AF did not decrease upon culture with the strain KTTM, the relationship between the AF degradation in the culture supernatant of the strain KTTM and the AF production-inhibitory activity of the strain appears weak. It might be important to clarify the relationship between cyclo(l-Ala-Gly) production and the AF production-inhibitory activity of KTTM to determine the mechanism underlying the strain’s strong inhibitory activity.

We previously found that cyclo(l-Ala-l-Pro) inhibited AfGST activity [19]. As fungal GST is believed to play a role in the response to oxidative stress, a key factor for AF production by aflatoxigenic fungi, we speculated that cyclo(l-Ala-L-Pro) would inhibit AF production by inhibiting AfGST activity. Although cyclo(l-Ala-Gly) and cyclo(l-Ala-L-Pro) commonly possess an l-Ala-containing diketopiperazine skeleton and inhibit AF production, cyclo(l-Ala-Gly) did not inhibit AfGST activity at the concentrations tested, suggesting that the mechanism by which cyclo(l-Ala-Gly) inhibits AF production does not involve AfGST inhibition. Research to identify the target molecule of cyclo(l-Ala-Gly) for AF production inhibition to clarify the mode of action of the compound and its congeners is currently ongoing.

## 4. Conclusions

The strain KTTM, identified as *K. aerogenes*, strongly inhibited the AF production by *A. flavus*, suggesting that the strain is a candidate agent for AF control. Cyclo(l-Ala-Gly) was isolated from the KTTM strain culture broth as an AF production inhibitor. The simple structure and strong AF production-inhibitory activity of cyclo(l-Ala-Gly) and its related compounds highlight their utility as AF control agents and biological probes for investigating the mechanisms of AF production by aflatoxigenic fungi.

## 5. Materials and Methods

### 5.1. Strains and Culture

*A. flavus* IFM 47798 was used as a producer of AFB_1_ and aflatoxin B_2_ (AFB_2_). The strain was cultured on potato dextrose agar medium (BD, Franklin Lakes, NJ, USA) at 25 °C for 2 weeks. A spore suspension was prepared from the culture at a concentration of 1.1 × 10^5^ CFU/µL and used as the stock and inoculum.

The strain KTTM was isolated as explained in Section 2.1. by a dilution method using a Bennet liquid medium consisting of glucose 1%, peptone 0.2%, meat extract 0.1%, and yeast extract 0.1%, with a pH of 7.2. KTTM was identified as *Krebsiella aerogenes* by TechnoSuruga Laboratory (Shizuoka, Japan). The strain was cultured at 27.5 °C on a rotary shaker (150 rpm) for 3 days in a Bennet liquid medium, and the culture broth was stored as a 20% glycerol stock at −80 °C. The number of bacterial cells was determined from the number of colony-forming units.

### 5.2. Assay Method in Liquid Culture

A sample water solution (100 µL), passed through a 0.25 µm filter, or a bacterial culture broth (5 µL), cultured in a Bennet liquid medium at 27.5 °C for 3 days, was added to the potato dextrose liquid medium (1.9 or 2.0 mL) in a well of a microplate (24 wells). A spore suspension of *A. flavus* (5 µL) was inoculated into the medium and incubated statically for 4 days at 25 °C. AFB_1_ was produced mainly under these culture conditions. A mixture of 100 µL of culture broth and 400 µL of water:acetonitrile (9:1, *v*/*v*) was filtered (MinisartRC4, Sartorius, Gottingen, Germany), and the amount of AFB_1_ involved in the filtrate was analyzed by HPLC on a 250 mm × 4.6 mm inner diameter Capcell pak C_18_ UG 120 column (Osaka Soda, Osaka, Japan) via the isocratic elution of acetonitrile:methanol:water (1:3:6, *v*/*v*/*v*) over 20 min. at a flow rate of 1.0 mL/min with fluorescence detection at 450 nm (excitation: 365 nm).

### 5.3. Assay Method Using Peanuts

Peanuts (Chiba-handachi) were grown from seedlings, harvested, and stored in their shells at −25 °C. After removing the shells and skins from the raw peanuts (each weighing approximately 1.2 g), the peanuts were autoclaved. After being dipped in the strain KTTM culture broth, cultured in a Bennet medium at 27.5 °C for 3 days, or dipped in a dilution series of the culture broth for a few seconds, each peanut was put into a well of a microplate (12 wells), inoculated with *A. flavus* spores (5 µL), and incubated statically for 30 days at 25 °C. AFB_1_ and AFB_2_ were produced under these culture conditions. Each peanut, including the fungal mycelia grown on the peanut, was crushed and extracted with 5 mL of water: acetonitrile (1:9, *v*/*v*). The supernatant, obtained by centrifugation, was passed through a cartridge for AF purification (Autoprep MF-A 1000, Resonac Co., Tokyo, Japan), and 1 mL of the pass-through solution was lyophilized. The residue was dissolved in 400 µL of water:acetonitrile (9:1, *v*/*v*) and filtered (MinisartRC4), and then the AFB_1_ and AFB_2_ contents in the solution were analyzed by HPLC as previously explained.

### 5.4. Isolation of the Active Component from the Strain KTTM

The bacterial glycerol stock (210 µL) was inoculated into a Bennett medium (7 mL) in a test tube and incubated at 27.5 °C for 2 days at 150 rpm. This preculture (3 mL) was transferred into a Bennett medium (100 mL) in a 500 mL Erlenmeyer flask, which was incubated at 27.5 °C for 5 days at 150 rpm.

Three liters of the strain KTTM culture broth were centrifuged at 5000× *g* for 10 min, and the culture supernatant was applied to a charcoal column (Activated Charcoal, FUJIFILM Wako Chemicals, 100 g, Osaka, Japan) packed with water and eluted with 500 mL of 10% ethanol after washing the column with 300 mL of water. The 10% ethanol eluate was concentrated to 18.5 mL under reduced pressure, and the concentrated solution was purified using HPLC on a 250 mm × 10 mm i.d. Capcell pak C_18_ column with a gradient of 0–100% CH_3_CN in water containing 0.1% TFA for 15 min at a flow rate of 2.5 mL/min by detection at 300 nm to obtain the active fraction (40.6 mg). The active fraction was further purified on a 250 mm × 4.6 mm i.d. Capcell pak C_18_ column with a gradient of 0–100% CH_3_CN in water containing 0.1% TFA for 15 min at a flow rate of 1.0 mL/min with detection at 220 nm to obtain the active fraction (4.0 mg), which was finally purified by HPLC on a 250 mm × 4.6 mm i.d. Senshu Pak DOCOSIL SP-100 column (Senshu Scientific, Tokyo, Japan) with the isocratic elution of water containing 10 mM CH_3_COONH_4_ at a flow rate of 1.0 mL/min with detection at 210 nm to obtain the active component (retention time: 5.2 min; 1.15 mg). The active component was identified as follows: ESI-Q/TOFMS *m*/*z* 129.0666 (M + H)^+^ (calcd for C_5_H_9_N_2_O_2_, 129.0664); [α]^27^_D_ − 11.5 (*c* = 0.06, H_2_O) [authentic cyclo(L-Ala-Gly) (Bachem, Bubendorf, Switzerland): [α]^27^_D_ − 13.8 (*c* = 0.06, H_2_O)]; δ_H_ (DMSO-*d*_6_, 500 MHz): 8.16 (1H, br.s, NH), 7.97 (1H, br.s, NH), 3.84(1H, q, *J* = 7 Hz), 2.72 (2H, m), 1.25 (3H, d, *J* = 7 Hz); and δ_C_ (DMSO-*d*_6_, 125 MHz): 168.9, 166.3, 49.7, 44.5, 18.7.

### 5.5. Preparation of the Cyclo(d-Ala-Gly), Cyclo(l-Abu(2)-Gly), Cyclo(Gly-Gly), Cyclo(l-Val-Gly), Cyclo(l-Nva-Gly), and Cyclo(l-Leu-Gly)

The diketopiperazines were prepared according to the method of Thajudeen et al. [24].

D-Ala-OMe-HCl (0.415 g, 3 mmol, Watanabe Chem. Inc., Ltd., Hiroshima, Japan) was dissolved in 3 mL of dry DMF. Boc-Gly-OH (0.526 g, 3 mmol, Watanabe Chem.), 2-(1*H*-benzotriazol-1-yl)-1,1,3,3-tetramethyluronium hexafluorophosphate (1.37 g, 3.6 mmol), and *N*,*N*-diisopropylethylamine (0.38 g, 6.0 mmol) were added to the solution, and the mixture was stirred at room temperature overnight. After adding ethyl acetate (100 mL) to the reaction solution, the solution was washed with 5% NaHCO_3_ (120 mL) and 5% NaCl (120 mL) and dried over anhydrous Na_2_SO_4_. After evaporation, the residue (0.451 g) was chromatographed on a silica gel column (Silica gel 60 (0.063–0.200 mm), 20 g, Merck, Darmstadt, Germany) by isocratic elution with *n*-hexane-EtOAc (1:1) to obtain the Boc-and OMe-protected dipeptide (0.296 g). The protected dipeptide (0.296 g) was deprotected and cyclized by autoclave at 121 °C for 4 h in water (30 mL). The water solution was evaporated, and the residue (0.238 g) was purified on a silica gel column for chromatography (Silica gel 60 (0.063–0.200 mm), 20 g, CH_2_Cl_2_: MeOH (9:1)) to obtain the cyclo(d-Ala-Gly) (42 mg). Similarly, 84 mg of cyclo(l-Abu(2)-Gly), 22 mg of cyclo(Gly-Gly), 106 mg of cyclo(l-Val-Gly), 66 mg of cyclo(l-Nva-Gly), and 137 mg of cyclo(l-Leu-Gly) were obtained from each 6 mmol of the Gly-OMe-HCl and Boc-l-Abu(2)-OH, Gly-OMe-HCl and Boc-Gly-OH, l-Val-OMe-HCl and Boc-Gly-OH, l-Nva-OMe-HCl and Boc-Gly-OH, and l-Leu-OMe-HCl and Boc-Gly-OH (Watanabe Chem.), respectively.

The cyclo(d-Ala-Gly) was identified as follows: ESI-Q/TOFMS *m*/*z* 129.0666 (M + H)^+^ (calcd for C_5_H_9_N_2_O_2_, 129.0664); [α]^27^_D_ + 3.7 (*c* = 0.26, H_2_O); δ_H_ (DMSO-*d*_6_, 500 MHz): 8.16 (1H, br.s, NH), 7.98 (1H, br.s, NH), 3.84 (1H, q, *J* = 7 Hz), 3.72 (2H, m), 1.25 (3H, d, *J* = 7 Hz); and δ_C_ (DMSO-*d*_6_, 125 MHz): 168.6, 166.3, 49.7, 44.5, 18.7.

The cyclo(l-Abu(2)-Gly) was identified as follows: ESI-Q/TOFMS *m*/*z* 143.0823 (M + H)^+^ (calcd for C_6_H_11_N_2_O_2_, 143.0821); [α]^27^_D_ +21.2 (*c* = 0.64, H_2_O); δ_H_ (DMSO-*d*_6_, 500 MHz): 8.17 (1H, br.s, NH), 8.00 (1H, br.s, NH), 3.77 (1H, d, *J* = 17 Hz), 3.72 (1H, m), 3.68 (1H, d, *J* = 17 Hz), 1.61–1.77 (2H, m), 0.85 (3H, t, *J* = 7 Hz); and δ_C_ (DMSO-*d*_6_, 125 MHz): 167.8, 166.1, 55.1, 44.3, 25.9, 8.8.

The cyclo(Gly-Gly) was identified as follows: ESI-Q/TOFMS *m*/*z* 115.0506 (M + H)^+^ (calcd for C_4_H_7_N_2_O_2_, 115.0508); δ_H_ (DMSO-*d*_6_, 500 MHz): 8.02 (1H, br.s, NH), 3.70 (2H); and δ_C_ (DMSO-*d*_6_, 125 MHz): 166.1, 44.3.

The cyclo(l-Val-Gly) was identified as follows: ESI-Q/TOFMS *m*/*z* 157.0974 (M + H)^+^ (calcd for C_7_H_13_N_2_O_2_, 157.0977); [α]^27^_D_ +26.4 (*c* = 0.21, H_2_O); δ_H_ (DMSO-*d*_6_, 500 MHz): 8.19 (1H, br.s, MH), 8.01 (1H, br.s, MH), 3.81 (1H, d, *J* = 18 Hz), 3.62 (1H, dd, *J* = 18, 3 Hz), 3.52 (1H, t, *J* = 3 Hz), 2.10 (1H, m), 0.92 (3H, d, *J* = 7 Hz), 0.85 (3H, d, *J* = 7 Hz); and δ_C_ (DMSO-*d*_6_, 125 MHz): 167.3, 166.1, 59.8, 44.1, 32.3, 18.6, 17.1.

The cyclo(l-Nva-Gly) was identified as follows: ESI-Q/TOFMS *m*/*z* 157.0975 (M + H)^+^ (calcd for C_7_H_13_N_2_O_2_, 157.0977); [α]^27^_D_ +19.9 (*c* = 0.21, H_2_O); δ_H_ (DMSO-*d*_6_, 500 MHz): 8.20 (1H, br.s, NH), 7.99 (1H, br.s, NH), 3.78 (1H, d, *J* = 18 Hz), 3.71 (1H, m), 3.65 (1H, dd, *J* = 18, 2 Hz), 1.57-1.69 (2H, m), 1.25–1.39 (2H, m), 0.87 (3H, t, *J* = 7 Hz); and δ_C_ (DMSO-*d*_6_, 125 MHz): 168.1, 166.2, 54.1, 44.3, 35.0, 17.4, 13.7.

The cyclo(l-Leu-Gly) was identified as follows: ESI-Q/TOFMS *m*/*z* 171.1134 (M + H)^+^ (calcd for C_8_H_15_N_2_O_2_, 171.1134); [α]^27^_D_ +25.1 (*c* = 0.19, H_2_O); δ_H_ (DMSO-*d*_6_, 500 MHz): 8.23 (1H, br.s, NH), 7.99 (1H, br.s, NH), 3.83 (1H, d, *J* = 17 Hz), 3.65 (1H, m), 3.61 (1H, dd, *J* = 17, 3 Hz), 1.76 (1H, m), 1.57 (2H, m), 0.89 (3H, d, *J* = 7 Hz), 0.86 (3H, d, *J* = 7 Hz); and δ_C_ (DMSO-*d*_6_, 125 MHz): 168.7, 166.4, 52.9, 44.3, 42.2, 23.6, 22.9, 21.8.

### 5.6. AF Degradation Activity of the Strain KTTM

The AF acetonitrile solution (50 or 100 µL, 2.5 ppm of AFB_1_) was placed in each well of the microplate (12 wells), and the plate was left in a safety cabinet for 1 h to remove the acetonitrile. A liquid Bennet medium (2 mL) with or without inoculation with the KTTM strain (5 µL of the culture broth cultured in a liquid Bennet medium for 3 days) or the KTTM strain supernatant of the culture broth (1 mL), centrifuged and passed through a 0.25 µm filter, was placed in each well (to a final concentration of AFB_1_: 0.125 ppm). After 3 days of incubation at 27.5 °C, the AFB_1_ amount in each well was measured according to the method described in Section 5.2.

### 5.7. Preparation of the Recombinant AfGST

Lyophilized *A. flavus* mycelia were ground using a FastPrep-24 instrument (MP Biomedicals, Irvine, CA, USA). The total RNA was extracted using Trizol reagent (Thermo Fisher Scientific, Waltham, MA, USA) and purified using a PureLink RNA Mini Kit (Thermo Fisher Scientific). Complementary DNA was synthesized using a ReverTra Ace qPCR RT Master Mix (TOYOBO, Osaka, Japan). The coding region of AfGST (AFLA_076480, in JCVI-afl1-v2.0) was PCR amplified using the primers AfGST-F-*Hind* III (5′-TATAATAATGAAGCTTTCTCTTAAGCCTATTATCCT-3′) and AfGST-R-*Kpn* I (5′-TTGTAGTCAGATCTGGTAGGATGCGTCATTTTCG-3′). The PCR product was cloned in the pT7-FLAG-2 expression vector (Sigma-Aldrich, St. Louis, MO, USA) using the *Hind*III and *Kpn*I restriction sites with an In-Fusion HD Cloning Kit (TaKaRa, Shiga, Japan). The resulting vector was amplified with *E. coli* DH5ɑ and introduced into *E. coli* BL21 (DE3). After IPTG induction (1 mM) at 37 °C for 6 h in LB medium, the bacterial cells (Sigma-Aldrich) containing DNase I (50 U) and lysozyme (0.1 mg/mL) were suspended in CelLytic™ B Cell Lysis Reagent and incubated for 30 min. After centrifugation, the supernatant containing the AfGST-FLAG was collected and purified using anti-FLAG M2 affinity gel (Sigma-Aldrich) according to the manufacturer’s instructions.

### 5.8. Measurement of the GST Activity of AfGST-FLAG

The GST activity was measured according to a previously reported method [19], with some modifications. Briefly, AfGST-FLAG (2 μg) in 100 μL of 50 mM Tris-HCl (pH 8.0) was incubated with ethacrynic acid or diketopiperazines and glutathione (GSH) at room temperature for 30 min. After incubation, 100 μL of 50 mM Tris-HCl (pH 8.0) containing 1-chloro-2,4-dinitrobenzene (CDNB) was added to the mixture, and the absorbance measurement at 340 nm was started immediately (0 min). The final concentrations of GSH and CDNB were 5 and 1 mM, respectively. The absorbance at 0 min was subtracted from that at 3 min to estimate the ΔA_340_/min. The ratio of the ΔA_340_/min was calculated using the control without chemicals as 1.

### 5.9. Statistical Analysis

Boxplots were generated using the ggplot2 package of R (https://www.r-project.org, accessed on 16 June 2023). The statistical differences of the treated groups compared to the controls were determined by ordinary one-way ANOVA followed by Dunnett’s test when the variances of all groups could be assumed to be equal and by Brown–Forsythe and Welch ANOVA followed by Dunnett’s T3 test when the variances could not be assumed to be equal. Statistical analyses were performed with GraphPad Prism 10 ver.10.1.1 (GraphPad Software, La Jolla, CA, USA).

## Figures and Tables

**Table 1:** IC_50_ values of cyclo(l-Ala-Gly) and related compounds for inhibiting AF production.

Compound	IC_50_ (mM)
cyclo(l-Ala-Gly)	0.75
cyclo(d-Ala-Gly)	>10
cyclo(l-Ala-L-Pro)	1.9
cyclo(l-Abu(2)-Gly)	0.01
cyclo(l-Nva-Gly)	0.09
cyclo(l-Val-Gly)	0.04
cyclo(l-Leu-Gly)	4.2
cyclo(Gly-Gly)	>5.0

## Data Availability

The data will be made available upon request.

## Supplementary Materials

The following supporting information can be downloaded at: https://www.mdpi.com/article/10.3390/toxins16030141/s1, Method: Analysis of the mycelial weight. Figure S1: Alignment of the partial sequences of 16S rDNA from the strain KTTM (query) and *Klebsiella aerogenes* NBRC 13534 (Sbjct). Figure S2: ^1^H NMR spectrum of the active component in DMSO-*d*_6_ (500 MHz). Figure S3: ^13^C NMR spectrum of the active component in DMSO-*d*_6_ (125 MHz). Figure S4: Effect of the strain KTTM on the growth of *A. flavus* on peanuts. Figure S5: Effects of cyclo(L-Ala-Gly) and related compounds on *A. flavus* glutathione-*S*-transferase activity.
