# Supplementary material for: Inhibition of Aflatoxin Production in Aspergillus flavus by a Klebsiella sp. and Its Metabolite Cyclo(l-Ala-Gly)
Source: Toxins (Basel). 2024 Mar 8;16(3):141. doi: 10.3390/toxins16030141 (PMC10973989; doi:10.3390/toxins16030141)
Supplement: Supplementary file 1 [file toxins-16-00141-s001.zip › toxins-2863718-supplementary.pdf]

Supplementary Materials: Inhibition of Aflatoxin Production in *Aspergillus flavus* by a *Klebsiella* sp. and Its Metabolite Cyclo(L-Ala-Gly)

Shohei Sakuda, Masaki Sunaoka, Maho Terada, Ayaka Sakoda, Natsumi Ishijima, Noriko Hakoshima, Kenichi Uchida, Hirofumi Enomoto and Tomohiro Furukawa

CONTENTS

Method

Analysis of mycelial weight

Figures

Figure S1. Alignment of the partial sequences of 16S rDNA from the strain KTTM (Query) and *Klebsiella aerogenes* NBRC 13534 (Sbjct)

Figure S2. ^{1}H NMR spectrum of the active component in DMSO-d_6 (500 MHz)

Figure S3. ^{13}C NMR spectrum of the active component in DMSO-d_6 (125 MHz)

Figure S4. Effect of the strain KTTM on the growth of *A. flavus* on peanuts

Figure S5. Effects of cyclo(L-Ala-Gly) and related compounds on *A. flavus* glutathione-*S*-transferase activity

Method

Analysis of the mycelial weight

Cyclo(L-Ala-Gly) water solution (100 μL), passed through a 0.25 μm filter, was added to potato dextrose liquid medium (1.9 mL) in a well of a microplate (24 wells). A spore suspension of *A. flavus* (5 μL, 1.1×10^5 CFU/μL) was inoculated into the medium and incubated statically for 4 days at 25 °C. The mycelia in each well were collected in a 1.5 mL microtube. After drying the mycelia at 50 °C for 4 days, the mycelial weights were calculated by subtracting the weight of a 1.5 mL microtube without mycelia from the total weight.

```
Query    1 ATTGAACGCTGGCGGCAGGCCTAACACATGCAAGTCGAGCGGTARCACAGAGAGCTTGCT   60
           |||||||||||||||||||||||||||||||||||||||||||| |||||||||||||||
Sbjct    1 ATTGAACGCTGGCGGCAGGCCTAACACATGCAAGTCGAGCGGTAGCACAGAGAGCTTGCT   60

Query   61 CTCGGGTGACGAGCGGCGGACGGGTGAGTAATGTCTGGGAAACTGCCTGATGGAGGGGGA  120
           ||||||||||||||||||||||||||||||||||||||||||||||||||||||||||||
Sbjct   61 CTCGGGTGACGAGCGGCGGACGGGTGAGTAATGTCTGGGAAACTGCCTGATGGAGGGGGA  120

Query  121 TAACTACTGGAAACGGTAGCTAATACCGCATAACGTCGCAAGACCAAAGTGGGGGACCTT  180
           ||||||||||||||||||||||||||||||||||||||||||||||||||||||||||||
Sbjct  121 TAACTACTGGAAACGGTAGCTAATACCGCATAACGTCGCAAGACCAAAGTGGGGGACCTT  180

Query  181 CGGGCCTCATGCCATCAGATGTGCCCAGATGGGATTAGCTAGTAGGTGGGGTAATGGCTC  240
           ||||||||||||||||||||||||||||||||||||||||||||||||||||||||||||
Sbjct  181 CGGGCCTCATGCCATCAGATGTGCCCAGATGGGATTAGCTAGTAGGTGGGGTAATGGCTC  240

Query  241 ACCTAGGCGACGATCCCTAGCTGGTCTGAGAGGATGACCAGCCACACTGGAACTGAGACA  300
           ||||||||||||||||||||||||||||||||||||||||||||||||||||||||||||
Sbjct  241 ACCTAGGCGACGATCCCTAGCTGGTCTGAGAGGATGACCAGCCACACTGGAACTGAGACA  300

Query  301 CGGTCCAGACTCCTACGGGAGGCAGCAGTGGGGAATATTGCACAATGGGCGCAAGCCTGA  360
           ||||||||||||||||||||||||||||||||||||||||||||||||||||||||||||
Sbjct  301 CGGTCCAGACTCCTACGGGAGGCAGCAGTGGGGAATATTGCACAATGGGCGCAAGCCTGA  360

Query  361 TGCAGCCATGCCGCGTGTATGAAGAAGGCCTTCGGGTTGTAAAGTACTTTCAGCGAGGAG  420
           ||||||||||||||||||||||||||||||||||||||||||||||||||||||||||||
Sbjct  361 TGCAGCCATGCCGCGTGTATGAAGAAGGCCTTCGGGTTGTAAAGTACTTTCAGCGAGGAG  420

Query  421 GAAGGCRTTAAGGTTAATAACCTTRGYGATTGACGTTACTCGCAGAAGAAGCACCGGCTA  480
           |||||| ||||||||||||||||| | |||||||||||||||||||||||||||||||||
Sbjct  421 GAAGGCGTTAAGGTTAATAACCTTGGCGATTGACGTTACTCGCAGAAGAAGCACCGGCTA  480

Query  481 ACTCCGTGCCAGCAGCCGCGGTAATACGGAGGGTGCAAGCGTTAATCGGAATTACTGGGC  540
           ||||||||||||||||||||||||||||||||||||||||||||||||||||||||||||
Sbjct  481 ACTCCGTGCCAGCAGCCGCGGTAATACGGAGGGTGCAAGCGTTAATCGGAATTACTGGGC  540

Query  541 GTAAAGCGCACGCAGGCGGTCTGTCAAGTCGGATGTGAAATCCCCGGGCTCAACCTGGGA  600
           ||||||||||||||||||||||||||||||||||||||||||||||||||||||||||||
Sbjct  541 GTAAAGCGCACGCAGGCGGTCTGTCAAGTCGGATGTGAAATCCCCGGGCTCAACCTGGGA  600

Query  601 ACTGCATTCGAAACTGGCAGGCTAGAGTCTTGTAGAGGGGGGTAGAATTCCAGGTGTAGC  660
           ||||||||||||||||||||||||||||||||||||||||||||||||||||||||||||
Sbjct  601 ACTGCATTCGAAACTGGCAGGCTAGAGTCTTGTAGAGGGGGGTAGAATTCCAGGTGTAGC  660

Query  661 GGTGAAATGCGTAGAGATCTGGAGGAATACCGGTGGCGAAGGCGGCCCCCTGGACAAAGA  720
           ||||||||||||||||||||||||||||||||||||||||||||||||||||||||||||
Sbjct  661 GGTGAAATGCGTAGAGATCTGGAGGAATACCGGTGGCGAAGGCGGCCCCCTGGACAAAGA  720

Query  721 CTGACGCTCAGGTGCGAAAGCGTGGGGAGCAAACAGGATTAGATACCCTGGTAGTCCACG  780
           ||||||||||||||||||||||||||||||||||||||||||||||||||||||||||||
Sbjct  721 CTGACGCTCAGGTGCGAAAGCGTGGGGAGCAAACAGGATTAGATACCCTGGTAGTCCACG  780
```

Figure S1. Alignment of the partial sequences of 16S rDNA from the strain KTTM (Query) and *Klebsiella aerogenes* NBRC 13534 (Sbjct)

```
Query   781 CCGTAAACGATGTCGACTTGGAGGTTGTGCCCTTGAGGCGTGGCTTCCGGAGCTAACGCG  840
            ||||||||||||||||||||||||||||||||||||||||||||||||||||||||||||
Sbjct   781 CCGTAAACGATGTCGACTTGGAGGTTGTGCCCTTGAGGCGTGGCTTCCGGAGCTAACGCG  840

Query   841 TTAAGTCGACCGCCTGGGGAGTACGGCCGCAAGGTTAAAACTCAAATGAATTGACGGGGG  900
            ||||||||||||||||||||||||||||||||||||||||||||||||||||||||||||
Sbjct   841 TTAAGTCGACCGCCTGGGGAGTACGGCCGCAAGGTTAAAACTCAAATGAATTGACGGGGG  900

Query   901 CCCGCACAAGCGGTGGAGCATGTGGTTTAATTCGATGCAACGCGAAGAACCTTACCTACT  960
            ||||||||||||||||||||||||||||||||||||||||||||||||||||||||||||
Sbjct   901 CCCGCACAAGCGGTGGAGCATGTGGTTTAATTCGATGCAACGCGAAGAACCTTACCTACT  960

Query   961 CTTGACATCCAGAGAACTTAGCAGAGATGCTTTGGTGCCTTCGGGAACTCTGAGACAGGT 1020
            ||||||||||||||||||||||||||||||||||||||||||||||||||||||||||||
Sbjct   961 CTTGACATCCAGAGAACTTAGCAGAGATGCTTTGGTGCCTTCGGGAACTCTGAGACAGGT 1020

Query  1021 GCTGCATGGCTGTCGTCAGCTCGTGTTGTGAAATGTTGGGTTAAGTCCCGCAACGAGCGC 1080
            ||||||||||||||||||||||||||||||||||||||||||||||||||||||||||||
Sbjct  1021 GCTGCATGGCTGTCGTCAGCTCGTGTTGTGAAATGTTGGGTTAAGTCCCGCAACGAGCGC 1080

Query  1081 AACCCTTATCCTTTGTTGCCAGCGGTYMGGCCGGGAACTCAAAGGAGACTGCCAGTGATA 1140
            ||||||||||||||||||||||||||  ||||||||||||||||||||||||||||||||
Sbjct  1081 AACCCTTATCCTTTGTTGCCAGCGGTNCGGCCGGGAACTCAAAGGAGACTGCCAGTGATA 1140

Query  1141 AACTGGAGGAAGGTGGGGATGACGTCAAGTCATCATGGCCCTTACGAGTAGGGCTACACA 1200
            ||||||||||||||||||||||||||||||||||||||||||||||||||||||||||||
Sbjct  1141 AACTGGAGGAAGGTGGGGATGACGTCAAGTCATCATGGCCCTTACGAGTAGGGCTACACA 1200

Query  1201 CGTGCTACAATGGCATATACAAAGAGAAGCGACCTCGCGAGAGCAAGCGGACCTCATAAA 1260
            ||||||||||||||||||||||||||||||||||||||||||||||||||||||||||||
Sbjct  1201 CGTGCTACAATGGCATATACAAAGAGAAGCGACCTCGCGAGAGCAAGCGGACCTCATAAA 1260

Query  1261 GTATGTCGTAGTCCGGATTGGAGTCTGCAACTCGACTCCATGAAGTCGGAATCGCTAGTA 1320
            ||||||||||||||||||||||||||||||||||||||||||||||||||||||||||||
Sbjct  1261 GTATGTCGTAGTCCGGATTGGAGTCTGCAACTCGACTCCATGAAGTCGGAATCGCTAGTA 1320

Query  1321 ATCGTAGATCAGAATGCTACGGTGAATACGTTCCCGGGCCTTGTACACACCGCCCGTCAC 1380
            ||||||||||||||||||||||||||||||||||||||||||||||||||||||||||||
Sbjct  1321 ATCGTAGATCAGAATGCTACGGTGAATACGTTCCCGGGCCTTGTACACACCGCCCGTCAC 1380

Query  1381 ACCATGGGAGTGGGTTGCAAAAGAAGTAGGTAGCTTAACCTTCGGGAGGGCGCTTACCAC 1440
            ||||||||||||||||||||||||||||||||||||||||||||||||||||||||||||
Sbjct  1381 ACCATGGGAGTGGGTTGCAAAAGAAGTAGGTAGCTTAACCTTCGGGAGGGCGCTTACCAC 1440

Query  1441 TTTGTGATTCATGACTGGGGTGAAG                                    1465
            |||||||||||||||||||||||||
Sbjct  1441 TTTGTGATTCATGACTGGGGTGAAG                                    1465
```

Figure S1. (continued) Alignment of the partial sequences of 16S rDNA from the strain KTTM (Query) and *Klebsiella aerogenes* NBRC 13534 (Sbjct)

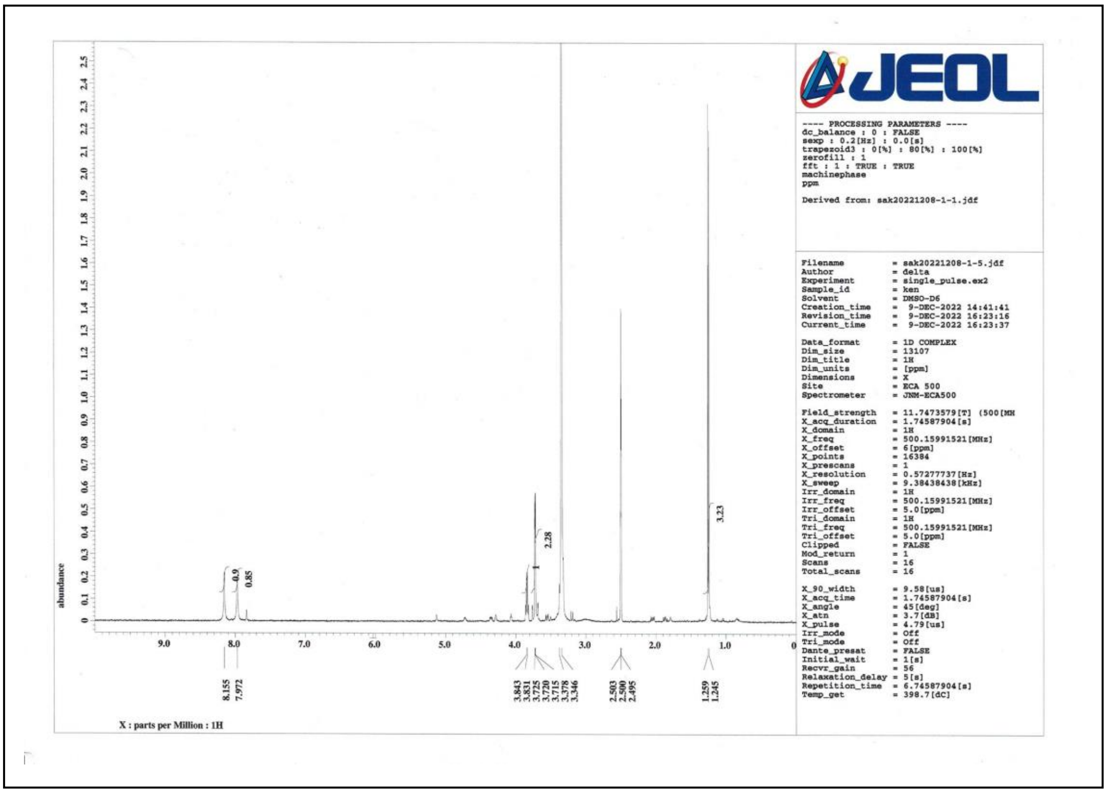

Figure S2. ^{1}H NMR spectrum of the active component in DMSO-d_6 (500 MHz)

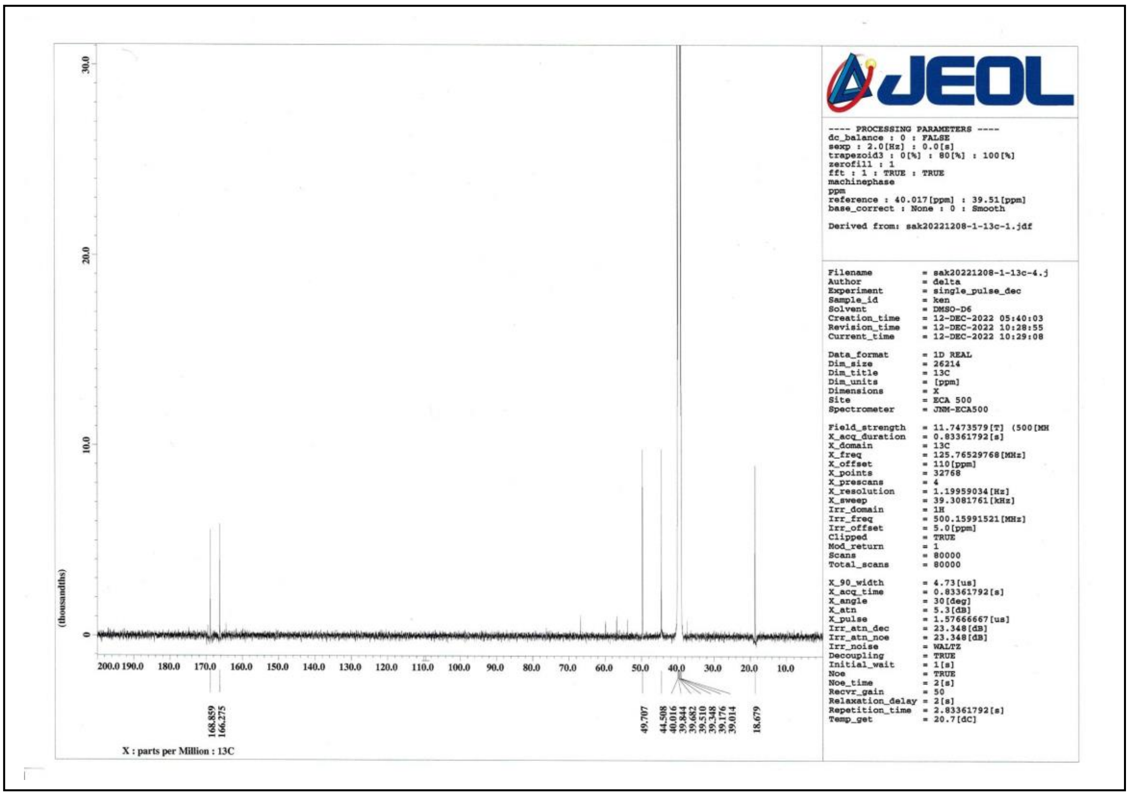

Figure S3. ^{13}C NMR spectrum of the active component in DMSO-d_6 (125 MHz)

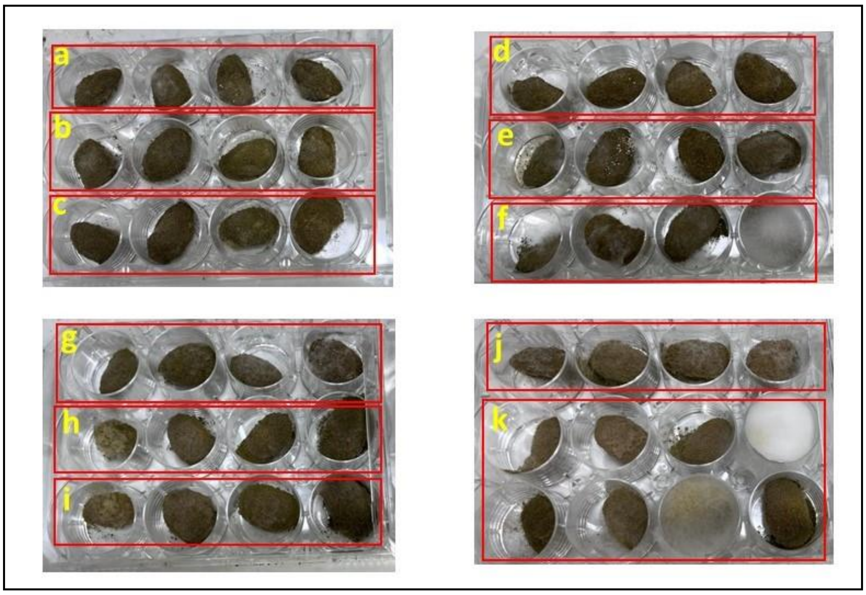

Figure S4. Effect of the strain KTTM on the growth of *A. flavus* on peanuts Autoclaved law peanuts without shells and skins were dipped for a few seconds in the culture broth of the strain KTTM (a, $6.2x10^8$ cells/mL) or a 10-time dilution series of dilutions of the culture broth (b, $6.2x10^7$ cells/mL; c, $6.2x10^6$ cells/mL; d, $6.2x10^5$ cells/mL; e, $6.2x10^4$ cells/mL; f, $6.2x10^3$ cells/mL; g, $6.2x10^2$ cells/mL; h, 6.2x10 cells/mL; i, 6.2 cells/mL; and j, <1 cell/mL), with a liquid Bennet medium used as the control (k), and incubated at 25 °C for 30 days.

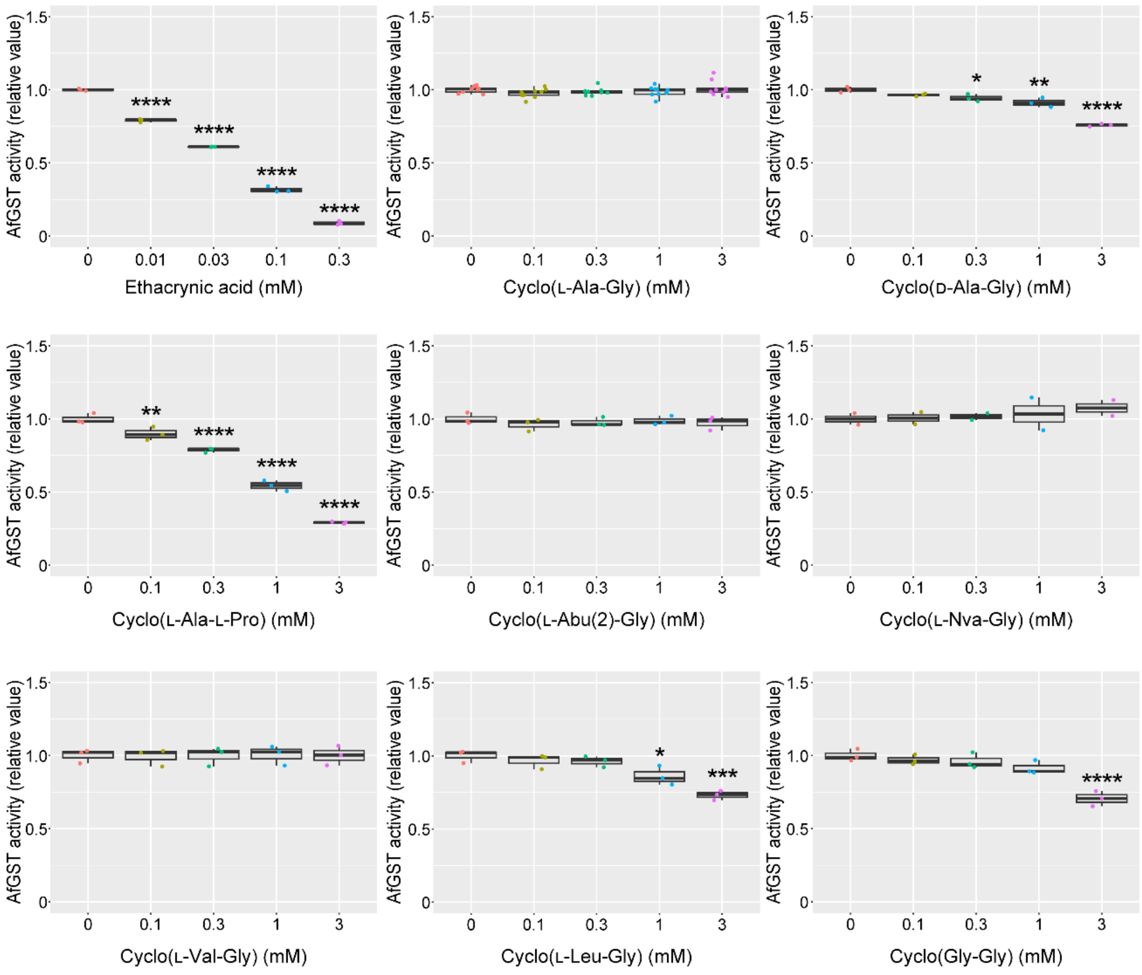

Figure S5. Effects of cyclo(L-Ala-Gly) and related compounds on *A. flavus* glutathione-*S*-transferase activity

Boxplots of the relative activity of *A. flavus* glutathione-*S*-transferase (AfGST) when treated with ethacrynic acid and diketopiperazines. The colored dots indicate individual values. n = 4. **p*<0.05, ***p*< 0.01, ****p*< 0.001, and *****p*< 0.0001 versus no added control, ordinary one-way ANOVA followed by Dunnett's test.
